# Supplementary material for: Is It Possible to Educate, Intervene or “Cure” Autism Spectrum Disorder? A Content Analysis of YouTube Videos
Source: Int J Environ Res Public Health. 2021 Feb 28;18(5):2350. doi: 10.3390/ijerph18052350 (PMC7967735; doi:10.3390/ijerph18052350)
Supplement: Supplementary file 1 [file ijerph-18-02350-s001.pdf]

Terms	URL	Duration	Number of views	Content	Number of likes	Number of dislikes	Understandability score	Actionability score
1	https://www.youtube.com/watch?v=iphBhcqqZh0	49	751215	4	17711	1412	90,91	66,67
1	https://www.youtube.com/watch?v=PtH2hx2CmUA	9	679659	4	10937	815	70,00	0,00
1	https://www.youtube.com/watch?v=hafw9f89NBI	6	523859	4	8955	390	60,00	33,33
1	https://www.youtube.com/watch?v=5USvp5A3Rew	5	119371	2	1319	2298	70,00	0,00
1	https://www.youtube.com/watch?v=MC5Szy-4RSc	75	116956	4	1671	132	72,73	100,00
1	https://www.youtube.com/watch?v=S4z1bZfbqM8	14	108633	4	3482	154	66,67	0,00
1	https://www.youtube.com/watch?v=uDmwjxYi5SE	2	78186	3	532	177	75,00	100,00
1	https://www.youtube.com/watch?v=d6l9xp0tUno	4	76992	3	379	38	77,78	0,00
1	https://www.youtube.com/watch?v=H58GGxDMIlI	9	72759	1	80	3	72,73	33,33
1	https://www.youtube.com/watch?v=BLqKWhLHB_o	12	72045	2	931	132	75,00	66,67
1	https://www.youtube.com/watch?v=FDL-a2IU144	10	71511	3	1090	120	80,00	100,00
1	https://www.youtube.com/watch?v=0CMSH_QvfMw	13	57093	3	3160	84	77,78	66,67
1	https://www.youtube.com/watch?v=8fsHiYFuaAk	4	56234	1	435	21	70,00	0,00
1	https://www.youtube.com/watch?v=9draWhFCtOg	10	47303	4	612	25	77,78	33,33
1	https://www.youtube.com/watch?v=MAcdwcCuR6A	12	37460	3	461	31	90,00	100,00
1	https://www.youtube.com/watch?v=451hJEMhjDM	7	29455	1	93	4	63,64	33,33
1	https://www.youtube.com/watch?v=3ojPiSJBtvM	2	20539	1	264	18	70,00	0,00
1	https://www.youtube.com/watch?v=5ys9XAkUi74	5	20069	1	204	12	66,67	0,00
1	https://www.youtube.com/watch?v=x20UOzqMlpI	5	17922	2	118	187	80,00	0,00
1	https://www.youtube.com/watch?v=exZVlbodN9A	17	17640	4	241	7	77,78	33,33
1	https://www.youtube.com/watch?v=MaDycaUTF10	2	16315	2	135	14	77,78	0,00
1	https://www.youtube.com/watch?v=KLtPxug_VbY	14	15927	2	490	203	90,91	100,00
1	https://www.youtube.com/watch?v=H19roctbP_c	12	14100	2	72	10	81,82	100,00
1	https://www.youtube.com/watch?v=UDa4PfjjWF0	5	11969	1	172	21	72,73	0,00
1	https://www.youtube.com/watch?v=pBh9vaY0uRc	2	9239	3	91	3	80,00	100,00
1	https://www.youtube.com/watch?v=gnK3hJDpzqc	3	7406	4	24	3	40,00	66,67
1	https://www.youtube.com/watch?v=b6qnMyosFmc	4	6411	2	51	278	66,67	0,00
1	https://www.youtube.com/watch?v=UJOSQW_u6Cc	50	6192	4	43	4	50,00	0,00
1	https://www.youtube.com/watch?v=z0Bcq2pf5VU	5	4783	1	71	6	77,78	66,67
1	https://www.youtube.com/watch?v=0S4OzFyamR4	12	4307	4	105	1	66,67	33,33
1	https://www.youtube.com/watch?v=475uk3haxPA	4	3710	1	16	6	55,56	0,00
1	https://www.youtube.com/watch?v=VpHQl4xtHy8	6	3193	2	228	14	33,33	66,67
1	https://www.youtube.com/watch?v=PID12nc2_FA	9	3061	4	166	5	70,00	33,33
1	https://www.youtube.com/watch?v=eJOkuQ-hOPM	12	2936	2	99	100	66,67	0,00
1	https://www.youtube.com/watch?v=rd6jn10gUqI	11	2887	2	20	2	66,67	0,00
1	https://www.youtube.com/watch?v=5dBy89qa_pU	2	2720	1	34	1	70,00	0,00

1	https://www.youtube.com/watch?v=nIemuvF8NbE	22	1759	2	45	14	55,56	0,00
1	https://www.youtube.com/watch?v=j_ia3J1_kTI	7	1502	4	20	3	77,78	33,33
1	https://www.youtube.com/watch?v=cVlH5qzhePM	2	1395	2	6	75	77,78	0,00
1	https://www.youtube.com/watch?v=6bjTOUAqdBo	10	1224	1	13	4	50,00	33,33
1	https://www.youtube.com/watch?v=W4etNc-pp74	11	1191	2	16	4	60,00	0,00
1	https://www.youtube.com/watch?v=cVdz2IP0ISE	11	1125	2	30	8	62,50	0,00
1	https://www.youtube.com/watch?v=QCE7rOlO0w0	4	1111	2	14	52	77,78	0,00
1	https://www.youtube.com/watch?v=jtP7ZcSS4QA	2	807	2	3	0	77,78	0,00
1	https://www.youtube.com/watch?v=2SArHS6-RlI	12	653	2	7	3	55,56	0,00
1	https://www.youtube.com/watch?v=4MFUI7f4t-c	10	637	4	47	1	75,00	0,00
1	https://www.youtube.com/watch?v=UaW8qOiHK4c	5	405	2	38	3	75,00	0,00
1	https://www.youtube.com/watch?v=jWfCBAbp5Us	3	393	5	17	1	44,44	0,00
1	https://www.youtube.com/watch?v=WBJfrjXc8NE	1	198	2	0	1	77,78	0,00
1	https://www.youtube.com/watch?v=0-e9LQf30J8	5	168	2	3	1	77,78	0,00
2	https://www.youtube.com/watch?v=uYEnco2QYOY	1	708944	6	47	4	77,78	66,67
2	https://www.youtube.com/watch?v=eoyY6MPOZS8	6	473560	1	1725	70	75,00	66,67
2	https://www.youtube.com/watch?v=RxzN8J34AEk	13	369427	1	9829	313	100,00	100,00
2	https://www.youtube.com/watch?v=L32A4HRo5SE	8	206974	2	1867	172	90,91	33,33
2	https://www.youtube.com/watch?v=vcbYDaOBrlQ	4	174234	1	2172	68	90,00	100,00
2	https://www.youtube.com/watch?v=rlOzbtxjDBw	4	117344	3	458	148	62,50	100,00
2	https://www.youtube.com/watch?v=AL7thHqhQ6g	16	67413	3	362	30	90,00	33,33
2	https://www.youtube.com/watch?v=rveXYcD24Go	101	28057	1	698	12	90,91	100,00
2	https://www.youtube.com/watch?v=L7u3YNnE878	7	25946	1	253	14	77,78	33,33
2	https://www.youtube.com/watch?v=bHX8LUv0-7I	2	21378	6	24	2	75,00	0,00
2	https://www.youtube.com/watch?v=GA0-F6RgvTE	5	21208	1	98	5	77,78	100,00
2	https://www.youtube.com/watch?v=sIjEKCzcw-E	13	21082	1	526	13	100,00	100,00
2	https://www.youtube.com/watch?v=vgz9_QwfoUk	44	16785	1	206	13	80,00	33,33
2	https://www.youtube.com/watch?v=EDprzYsYih8	5	15355	1	130	10	77,78	66,67
2	https://www.youtube.com/watch?v=-bNZzfLIzLI	3	14821	5	192	1	88,89	100,00
2	https://www.youtube.com/watch?v=DMV_HQ4a4_c	26	14520	6	135	6	80,00	100,00
2	https://www.youtube.com/watch?v=fRQBhSKBzkM	3	13798	3	89	3	77,78	33,33
2	https://www.youtube.com/watch?v=7qabSbwVBs4	2	13135	3	145	2	75,00	33,33
2	https://www.youtube.com/watch?v=54aCWAJfxnA	4	11658	1	154	3	88,89	100,00
2	https://www.youtube.com/watch?v=V_ZCOHSo0eI	243	9901	1	422	7	83,33	100,00
2	https://www.youtube.com/watch?v=bskwZqMT7l8	28	9360	1	41	3	90,00	66,67
2	https://www.youtube.com/watch?v=Q9LARxU5w6Q	3	8849	1	88	3	88,89	100,00
2	https://www.youtube.com/watch?v=2DWCY130P8w	9	8792	6	65	6	77,78	66,67
2	https://www.youtube.com/watch?v=dR60sEFFzbA	6	7877	2	42	4	77,78	100,00
2	https://www.youtube.com/watch?v=U1SekVKakWk	262	7506	1	271	11	91,67	100,00

2	https://www.youtube.com/watch?v=86NN0e5Wt14	101	6982	1	150	3	77,78	33,33
2	https://www.youtube.com/watch?v=LNt0TVfBLZs	6	6528	6	7	1	77,78	0,00
2	https://www.youtube.com/watch?v=koGyJXYXap8	2	6148	5	59	2	77,78	0,00
2	https://www.youtube.com/watch?v=zJddbTBWjkA	100	5255	5	288	1	66,67	0,00
2	https://www.youtube.com/watch?v=kHp8pAoR6lg	10	5514	1	59	2	77,78	0,00
2	https://www.youtube.com/watch?v=KiOnJQG8jjw	10	5116	1	182	10	90,00	66,67
2	https://www.youtube.com/watch?v=j-YWRnvY9B0	4	4890	3	83	1	77,78	0,00
2	https://www.youtube.com/watch?v=rd40AV8udRw	1	3997	3	44	3	77,78	0,00
2	https://www.youtube.com/watch?v=71CEJujXt-A	5	3870	6	40	0	77,78	0,00
2	https://www.youtube.com/watch?v=McdE1gvETcs	84	3822	5	180	0	60,00	0,00
2	https://www.youtube.com/watch?v=Q_mGYrJB-gg	78	3286	5	140	2	60,00	0,00
2	https://www.youtube.com/watch?v=b4VYXUwrycQ	108	3093	5	104	0	66,67	0,00
2	https://www.youtube.com/watch?v=x0GhS1HuWzU	10	2864	5	46	0	77,78	66,67
2	https://www.youtube.com/watch?v=XRNv9wNwwW4	2	2741	3	14	1	75,00	0,00
2	https://www.youtube.com/watch?v=AY3nLsARR28	29	2726	1	25	1	66,67	100,00
2	https://www.youtube.com/watch?v=AiE7B8yWcO8	130	2724	5	104	0	77,78	66,67
2	https://www.youtube.com/watch?v=boHRw88zNhw	120	2721	5	91	3	77,78	66,67
2	https://www.youtube.com/watch?v=yQd2jwQvPdw	11	2652	6	245	3	75,00	33,33
2	https://www.youtube.com/watch?v=suq_VqJ9teI	115	2642	5	88	7	77,78	66,67
2	https://www.youtube.com/watch?v=K2JVdzC2uFE	2	2480	6	38	0	66,67	66,67
2	https://www.youtube.com/watch?v=-9lvHcWBhck	117	2321	1	98	2	77,78	66,67
2	https://www.youtube.com/watch?v=qj6D0siD3e8	2	2301	1	6	0	77,78	0,00
2	https://www.youtube.com/watch?v=lrwTLRq1tXw	121	2261	1	81	1	77,78	66,67
2	https://www.youtube.com/watch?v=volDnHYWQH8	2	2197	1	11	1	77,78	0,00
2	https://www.youtube.com/watch?v=xZ3hoFb4zAI	3	2185	5	18	0	77,78	0,00
3	https://www.youtube.com/watch?v=ePTpO1aT-vY	4	150347	3	494	36	77,78	33,33
3	https://www.youtube.com/watch?v=VVSoL0fujJk	10	108792	1	1440	101	90,00	100,00
3	https://www.youtube.com/watch?v=r0-_c-Pj3HE	43	99426	1	1244	75	77,78	33,33
3	https://www.youtube.com/watch?v=i1ML7LuZj6k	10	73317	1	2002	68	77,78	66,67
3	https://www.youtube.com/watch?v=oUuY-awcg-M	40	43318	1	527	35	77,78	33,33
3	https://www.youtube.com/watch?v=MAcdwcCuR6A	12	37460	1	461	31	90,00	100,00
3	https://www.youtube.com/watch?v=9AnSAOiEkyc	9	28747	1	201	5	77,78	33,33
3	https://www.youtube.com/watch?v=rveXYcD24Go	101	28057	1	698	12	90,91	100,00
3	https://www.youtube.com/watch?v=L7u3YNnE878	7	25946	1	253	14	77,78	33,33
3	https://www.youtube.com/watch?v=ZWfif5AOvLE	17	21203	1	228	16	77,78	33,33
3	https://www.youtube.com/watch?v=vgz9_QwfoUk	44	16785	1	206	13	80,00	33,33
3	https://www.youtube.com/watch?v=hJ0_9E6t7Ek	27	13695	1	427	7	80,00	100,00
3	https://www.youtube.com/watch?v=8R0IDkIkdiA	29	13536	1	171	3	77,78	33,33
3	https://www.youtube.com/watch?v=BSIpW5ikZiU	30	12871	1	144	7	90,00	33,33

3	https://www.youtube.com/watch?v=rVrBkfeU--U	5	12332	1	93	6	88,89	66,67
3	https://www.youtube.com/watch?v=3uAVR_fgd0I	14	9204	1	637	7	90,00	66,67
3	https://www.youtube.com/watch?v=k4S-3viPabU	20	8648	1	122	3	80,00	66,67
3	https://www.youtube.com/watch?v=EWc-XDvZj0U	20	7320	1	141	2	100,00	66,67
3	https://www.youtube.com/watch?v=_hNVqkgl0mc	3	6937	1	29	3	88,89	0,00
3	https://www.youtube.com/watch?v=msrhApKcOQE	1	5963	1	15	0	75,00	33,33
3	https://www.youtube.com/watch?v=VNsIQd_FWuU	21	5144	1	52	0	77,78	33,33
3	https://www.youtube.com/watch?v=ouet3-BTPYw	18	5085	1	64	2	77,78	33,33
3	https://www.youtube.com/watch?v=8sf61Iw11LE	79	4827	1	70	1	90,91	50,00
3	https://www.youtube.com/watch?v=U8AZkKcQ9PU	45	4312	5	68	4	81,82	66,67
3	https://www.youtube.com/watch?v=V9cJMF2z9E4	9	3992	1	55	1	90,00	33,33
3	https://www.youtube.com/watch?v=71CEJujXt-A	5	3878	6	40	0	77,78	0,00
3	https://www.youtube.com/watch?v=oaOmUuytFak	82	3890	1	80	6	77,78	66,67
3	https://www.youtube.com/watch?v=Igj-VdMjLpM	4	3614	1	39	1	75,00	0,00
3	https://www.youtube.com/watch?v=vPhdFhwF2aE	87	3661	1	163	9	77,78	100,00
3	https://www.youtube.com/watch?v=OEqfRUaQ8dY	69	3122	5	78	4	77,78	0,00
3	https://www.youtube.com/watch?v=1myVtxhU0Is	4	3025	1	12	0	66,67	0,00
3	https://www.youtube.com/watch?v=AfDFQl9Wf2U	64	2935	1	64	1	71,43	66,67
3	https://www.youtube.com/watch?v=JR8ges_Vl8w	5	2910	1	40	0	62,50	0,00
3	https://www.youtube.com/watch?v=PILSH00l-qI	18	2906	1	36	0	71,43	0,00
3	https://www.youtube.com/watch?v=xIqCKTF-oko	61	2787	1	59	2	80,00	0,00
3	https://www.youtube.com/watch?v=53kUVvOOn4E	5	2718	1	48	0	77,78	66,67
3	https://www.youtube.com/watch?v=chocFB1IcYM	26	2606	1	21	4	77,78	100,00
3	https://www.youtube.com/watch?v=yKdKTAcPlb0	27	2547	1	35	2	77,78	66,67
3	https://www.youtube.com/watch?v=wntQtqXHyQo	9	2419	1	26	2	77,78	66,67
3	https://www.youtube.com/watch?v=Ju8VS5Snrpg	5	2303	1	38	5	77,78	33,33
3	https://www.youtube.com/watch?v=_3HPBEyA3uY	14	2289	1	53	1	71,43	0,00
3	https://www.youtube.com/watch?v=4bFTtNrzZFE	3	2256	1	19	1	77,78	0,00
3	https://www.youtube.com/watch?v=veq7emFEg-M	23	2229	1	25	3	75,00	0,00
3	https://www.youtube.com/watch?v=sYn4VutgLX4&fr	32	2155	1	23	1	71,43	33,33
3	https://www.youtube.com/watch?v=2zUMXxM2ZNU	25	2131	1	15	2	77,78	0,00
3	https://www.youtube.com/watch?v=u8YgkvzrI2I	6	2005	1	4	0	77,78	33,33
3	https://www.youtube.com/watch?v=_8dRP9uHDZQ	4	1931	1	18	0	66,67	33,33
3	https://www.youtube.com/watch?v=aMwula90x4s	36	1846	1	19	1	77,78	0,00
3	https://www.youtube.com/watch?v=aW8c27q8BrM	82	1744	1	45	0	77,78	100,00
3	https://www.youtube.com/watch?v=No2PNBMO-7I	9	1622	3	18	0	60,00	33,33

1=CURE<br>2=EDUCATION<br>3=INTERVENTION		Minutes	Number of views	1= Conventional interventions (CON)<br>2= Denying harmful or dangerous treatments (DEN)<br>3= Non-harmful alternative interventions (ALT)<br>4= Harmful therapies (HARM)<br>5= Information about ASD (INF)<br>6= Right to inclusive education (EDU)	Number of likes	Number of dislikes	Understandability score	Actionability score
